# Evaluation of Genotypic and Phenotypic Protease Virulence Tests for Dichelobacter nodosus Infection in Sheep

**DOI:** 10.1128/JCM.02403-16

**Published:** 2017-04-25

**Authors:** Andrew S. McPherson, Om P. Dhungyel, Richard J. Whittington

**Affiliations:** Farm Animal Health, Sydney School of Veterinary Science and School of Life and Environmental Sciences, The University of Sydney, Sydney, Australia; University of Tennessee

**Keywords:** AprV2, Australia, diagnosis, elastase, footrot, protease, sheep, qPCR

## Abstract

Dichelobacter nodosus is a fastidious, strictly anaerobic bacterium, an obligate parasite of the ruminant hoof, and the essential causative agent of virulent ovine footrot. The clinical disease results from a complex interplay between the pathogen, the environment, and the host. Sheep flocks diagnosed with virulent but not benign footrot in Australia may be quarantined and required to undergo a compulsory eradication program, with costs met by the farmer. Virulence of D. nodosus at least partially depends on the elaboration of a protease encoded by *aprV2* and manifests as elastase activity. Laboratory virulence tests are used to assist diagnosis because clinical differentiation of virulent and benign footrot can be challenging during the early stages of disease or when the disease is not fully expressed due to unfavorable pasture conditions. Using samples collected from foot lesions from 960 sheep from 40 flocks in four different geographic regions, we evaluated the analytical characteristics of qPCR tests for the protease gene alleles *aprV2* and *aprB2*, and compared these with results from phenotypic protease (elastase and gelatin gel) tests. There was a low level of agreement between clinical diagnosis and quantitative PCR (qPCR) test outcomes at both the flock and sample levels and poor agreement between qPCR test outcomes and the results of phenotypic virulence tests. The diagnostic specificity of the qPCR test was low at both the flock and individual swab levels (31.3% and 18.8%, respectively). By contrast, agreement between the elastase test and clinical diagnosis was high at both the flock level (diagnostic sensitivity [DSe], 100%; diagnostic specificity [DSp], 78.6%) and the isolate level (DSe, 69.5%; DSp, 80.5%).

## INTRODUCTION

Dichelobacter nodosus (formerly Fusiformis nodosus, Bacteroides nodosus) (1, 2), is a fastidious, strictly anaerobic bacterium and an obligate parasite of the ruminant hoof (1). It is the essential causative agent of virulent ovine footrot. This is a major economic and animal welfare burden for sheep farmers in Australia (3) and many other sheep-producing countries, including the United Kingdom and the United States (4, 5). Clinical footrot is the result of a complex interplay between the pathogen, the environment, and the host. Depending on the inherent susceptibility of the host, an interaction between strains of D. nodosus and other microbial flora on the skin may induce dermatitis and degradation of the hoof, following environmental predisposition. Footrot initially presents as a mild dermatitis of the interdigital skin and can progress in susceptible individuals to separation of the sole and horn of the hoof (“underrunning”) encouraged by warm (average daily temperature ≥10°C [50°F]) moist environmental conditions (6, 7). For descriptive and classification purposes, two clinical forms of footrot are recognized by Australian regulatory authorities, namely, virulent and benign (8). The clinical severity of an outbreak of footrot is partially determined by the virulence of the infecting D. nodosus strain(s), which are also classified as virulent or benign according to their *in vitro* phenotypic characteristics (8), but in reality there is a continuum, which includes intermediate forms (7). Virulence of D. nodosus at least partially depends on the elaboration of a protease encoded by *aprV2* that manifests as elastase activity (9).

Footrot lesions are graded using a simple scoring system ranging from 0 (clinically healthy) to 4 (severe underrunning of the hard horn of the hoof) (10). Score 3 and score 4 lesions are regarded severe and cause lameness; however, differentiation of virulent and benign outbreaks of footrot has been based on the prevalence of score 4 lesions (8, 11). Clinically virulent footrot is characterized by a high prevalence of sheep with score 4 lesions (≥10%), while score 4 lesions are rare (<1%) in outbreaks of clinically benign footrot (8, 11). Importantly, this classification scheme acknowledges that virulent and benign strains of D. nodosus are both capable of inducing severe lesions in a proportion of susceptible sheep under favorable environmental conditions.

Clinical differentiation of virulent and benign footrot is relatively straightforward when the disease is fully expressed; however, it can be difficult during the initial stages of infection or where environmental conditions do not enable the full expression of the disease (8, 11). In such circumstances, laboratory identification and virulence testing of the infecting D. nodosus strain(s) are used to assist diagnosis. Currently, virulent and benign strains of D. nodosus are differentiated using one or more phenotypic tests for proteolytic enzyme activity (12, 13). The elastase test, which measures temporal and quantitative variations in activity of the extracellular proteases expressed by virulent and benign D. nodosus strains (13), has been shown to correlate well with clinical diagnoses (14, 15). The gelatin gel test, which measures the differences in the thermostability of extracellular proteases between virulent and benign D. nodosus strains (12), is also used by Australian regulatory authorities but can be unreliable (14–16).

Phenotypic tests require microbiological culture of D. nodosus, a process that is expensive and laborious, requiring specialized equipment and training (8). Furthermore, there is evidence of discrepancies between the phenotypic virulence tests and clinical observations, with phenotypically virulent D. nodosus strains isolated from clinically benign outbreaks of footrot (13, 15, 16). These discrepancies may reflect the lack of reproducibility of the tests themselves, which are sensitive to culture conditions (12, 13), or the influences of other host, pathogen, or environmental factors on the expression of the disease. There is also evidence of discrepancies between the different phenotypic virulence tests and between these and a genotypic marker for virulence known as *intA* (15).

Virulent strains of D. nodosus secrete three subtilisin-like extracellular proteases, namely, acidic protease isoenzyme 2 (AprV2), acidic protease isoenzyme 5 (AprV5), and a basic protease (BprV), encoded by the genes *aprV2*, *aprV5*, and *bprV*, respectively (17, 18). Benign strains secrete the analogous proteases AprB2, AprB5, and BprB, encoded by the genes *aprB2*, *aprB5*, and *bprB*, respectively (17–19). Kennan et al. (9) reported that AprV2 is essential for virulence *in vivo* through the construction of an *aprV2* gene mutant of virulent D. nodosus strain VSC1703A and established that the elastase activity of AprV2 forms the basis of the elastase test. Sequence analysis of *aprV2* and its benign ortholog *aprB2* has shown that the two alleles differ by a two base-pair substitution (TA/CG) (9, 20). Recently, two quantitative real-time PCR (qPCR) tests targeting this substitution were developed in Europe (21, 22). Both tests were reported to identify D. nodosus and differentiate virulent and benign strains. This has been the subject of rural media interest following press releases from the Departments of Primary Industries (23, 24).

In Australia, the means by which outbreaks of footrot are classified by regulatory authorities as virulent or benign differs between states. In NSW, for example, the diagnosis of virulent footrot is primarily based on clinical examination; laboratory tests may be used to assist with a diagnosis, but cannot be the sole basis of a diagnosis (8, 25). However, in Western Australia, the diagnosis of virulent footrot is based entirely on the results of laboratory virulence testing, irrespective of the clinical severity of an outbreak (25). Furthermore, although virulent footrot is a notifiable disease in some states, legislative approaches and the means by which footrot is controlled vary. In NSW, for example, flocks with clinically virulent footrot are quarantined and must undergo a compulsory eradication program, with costs met by the farmer. Allworth (26) estimated that the cost of eradicating virulent footrot from a flock can exceed $10 per head per annum. Benign footrot is not considered amendable to control (see Discussion).

To declare the protease gene-based qPCR tests as being suitable for use in Australian diagnostic laboratories, they must be evaluated using reference samples collected from representative Australian sheep flocks, using the appropriate case definitions (11). The aim of this study was to subject these tests to the important initial steps in the validation pathway outlined in chapter 1.1.6 of the *Manual of Diagnostic Tests and Vaccines for Terrestrial Animals* (27), which defines agreed international standards, including comparisons with clinical diagnosis and the currently used phenotypic protease virulence tests.

## RESULTS

### Flock selection and clinical diagnosis.

**FIG 1:**
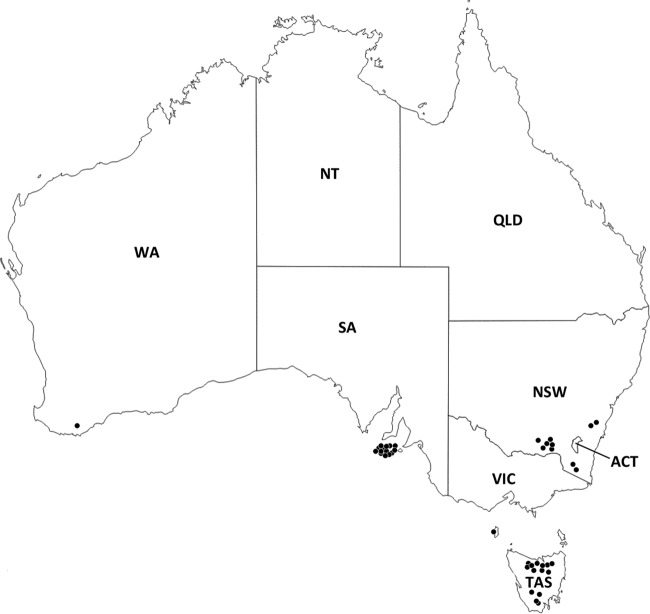
Distribution of Australian sheep flocks sampled in this present study. WA, Western Australia; NT, Northern Territory; SA, South Australia; QLD, Queensland; NSW, New South Wales; ACT, Australian Capital Territory; VIC, Victoria; TAS, Tasmania. Source: http://www.d-maps.com/carte.php?num_car=3293&lang=en.

**FIG 2:**
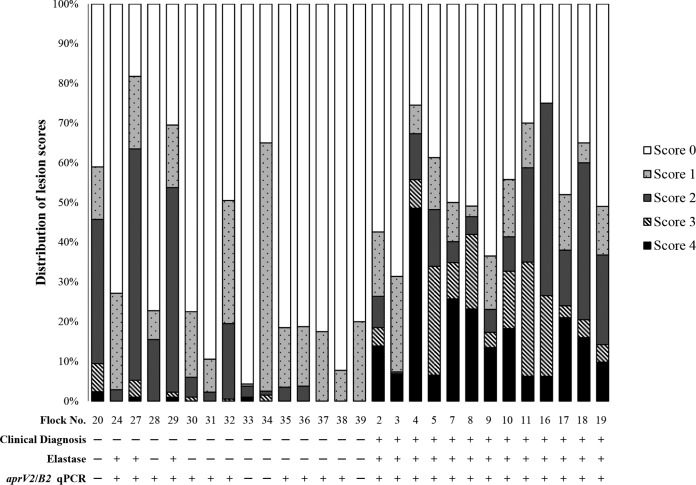
Summary of the proportions of feet with each lesion score for 28 of the flocks included in this study. Flocks were grouped according to clinical diagnosis. Lesion score summaries were not provided for 13 flocks inspected by animal health officers using method 3 (see Table 1). The number of sheep inspected in each flock is indicated in Table 1. Lesion scores were ordinal and based on Egerton and Roberts (10): clinically healthy feet were given a score of 0; mild lesions restricted to the interdigital skin were given a score of 1; if severe, a score of 2 was given; where underrunning of the posterior sole and soft horn of the heel were observed, a score of 3 was given; if the underrunning extended to the abaxial wall, a score of 4 was given. Flock-level clinical, elastase, and *aprV2*/*B2* qPCR diagnoses of virulent (**+**) and benign (—) footrot are also provided.

Forty Australian sheep flocks were selected for this study, including 24 flocks with clinically virulent footrot and 16 flocks with clinically benign footrot (Table 1). The flocks were selected from target populations in southeastern and southwestern Australia (Fig. 1). Three approaches to clinical diagnosis were used (Table 1). A summary of lesion scores was available for 28 flocks; the distribution of lesion scores observed in each of these flocks is illustrated in Fig. 2. Lesion swabs were collected for direct testing from 40 flocks, but lesion swabs for microbiological culture were collected from only 38 flocks.

**TABLE 1:** Details of the Merino flocks sampled during this study

Farm	State	Operator	Diagnostic approach	Season at time of inspection	No. of mobs inspected	No. of sheep inspected	No. or percentage of sheep with score 4 lesions	Clinical diagnosis	No. of sheep with lesions sampled	No. of swabs tested directly (*n* = 758)	No. of isolates collected (*n* = 469)
1	SA	AHO^*a*^	2	Winter	1	NA^*b*^	≥10%	Virulent	11	12	12
2	NSW	Authors	3	Winter	NA	54	23	Virulent	14	4	12
3	TAS	Authors	3	Winter	NA	51	11	Virulent	24	11	11
4	TAS	Authors	3	Winter	NA	52	40	Virulent	50	10	14
5	TAS	AHO	3	Winter	NA	42	10	Virulent	20	23	23
6	TAS	AHO	2	Winter	1	NA	≥10%	Virulent	20	23	23
7	TAS	AHO	3	Winter	NA	33	26	Virulent	20	20	20
8	TAS	AHO	3	Winter	NA	28	18	Virulent	20	19	19
9	TAS	AHO	3	Winter	NA	13	7	Virulent	13	13	13
10	TAS	AHO	3	Spring	NA	26	11	Virulent	20	15	15
11	TAS	AHO	3	Spring	NA	20	5	Virulent	20	8	8
12	SA	AHO	2	Spring	1	NA	≥10%	Virulent	10	10	10
13	SA	AHO	2	Spring	1	NA	≥10%	Virulent	6	9	9
14	SA	AHO	2	Spring	1	NA	≥10%	Virulent	7	8	7
15	SA	AHO	2	Spring	1	NA	≥10%	Virulent	10	13	13
16	SA	AHO	3	Spring	NA	16	3	Virulent	16	16	16
17	TAS	Authors	3	Spring	NA	25	14	Virulent	25	15	15
18	TAS	Authors	3	Spring	NA	50	19	Virulent	50	29	29
19	NSW	Authors	3	Summer	NA	51	15	Virulent	50	15	15
20	NSW	AHO	3	Summer	NA	50	4	Benign	14	14	14
21	TAS	AHO	2	Summer	1	NA	≥10%	Virulent	11	11	11
22	TAS	AHO	2	Summer	1	NA	≥10%	Virulent	17	17	17
23	SA	AHO	2	Summer	1	NA	≥10%	Virulent	4	4	4
24	NSW	Authors	3	Autumn	NA	20	0	Benign	20	20	21
25	NSW	Authors	3	Winter	NA	NA	≥10%	Virulent	10	14	14
26	TAS	AHO	2	Winter	1	NA	≥10%	Virulent	12	5	5
27	SA	Authors	1	Spring	1	100	4	Benign	40	40	20
28	SA	Authors	1	Spring	1	100	0	Benign	40	40	11
29	SA	Authors	1	Spring	1	100	2	Benign	40	40	12
30	SA	Authors	1	Spring	1	100	0	Benign	40	40	6
31	SA	Authors	1	Spring	1	170	0	Benign	40	40	3
32	SA	Authors	1	Spring	1	100	0	Benign	40	40	7
33	SA	AHO	2	Spring	1	1716	42	Benign	50	0	15
34	TAS	Authors	1	Winter	2	100	0	Benign	30	30	6
35	NSW	Authors	1	Winter	2	120	0	Benign	21	21	10
36	NSW	Authors	1	Winter	1	100	0	Benign	28	20	6
37	NSW	Authors	1	Winter	1	100	0	Benign	22	20	1
38	NSW	Authors	1	Winter	1	100	0	Benign	20	20	2
39	NSW	Authors	1	Winter	1	100	0	Benign	25	23	0
40	WA	AHO	2	Winter	1	NA	0	Benign	30	27	0

a AHO, animal health officer.

b NA, not available.

### Analytical characteristics of the qPCR tests.

Amplification efficiencies for *aprV2* and *aprB2* were 90.14 and 88.4, respectively, using the assay developed by Frosth et al. (21). Amplification efficiencies for *aprV2* and *aprB2* were 87.8 and 91.4, respectively, using the assay developed by Stäuble et al. (22). The limits of detection (LOD) of both qPCR tests for *aprV2* and *aprB2* were 0.005 and 0.05 pg, respectively.

Both assays were specific for the *aprV2* and *aprB2* alleles, and no amplification occurred for the 15 other bacterial species tested (Table 2).

**TABLE 2:** Analytical specificities of the *aprV2*/*V2* qPCR tests^*a*^

Species or strain	Location	Host	ID no.^*b*^	qPCR test developed by:			
				Stäuble et al. (22)		Frosth et al. (21)	
				*aprV2*	*aprB2*	*aprV2*	*aprB2*
Cardiobacterium hominis	NSW, Australia	Human	FD-3235	—	—	—	—
Corynebacterium ovis	NSW, Australia	Ovine	FD-2798	—	—	—	—
Dermatophilus congolensis	NSW, Australia	Ovine	FD-2839	—	—	—	—
Enterococcus uberis	NSW, Australia	Bovine	NA	—	—	—	—
Erysipelothrix rhusiopathiae	QLD, Australia	Ovine	FD-2825	—	—	—	—
Escherichia coli	NSW, Australia	Ovine	FD-2669	—	—	—	—
Fusobacterium necrophorum	NSW, Australia	Ovine	FD-2842	—	—	—	—
Klebsiella spp.	NSW, Australia	Bovine	NA	—	—	—	—
Moraxella bovis	NSW, Australia	Bovine	FD-2574	—	—	—	—
Nocardia spp.	NSW, Australia	Bovine	15-166	—	—	—	—
Pseudomonas aeruginosa	NSW, Australia	Ovine	FD-2696	—	—	—	—
Salmonella enterica serovar Typhimurium	VIC, Australia	Porcine	NA	—	—	—	—
Staphylococcus aureus	NSW, Australia	Bovine	2793	—	—	—	—
Streptococcus B	NSW, Australia	Ovine	2438	—	—	—	—
Suttonella indologenes	NSW, Australia	Human	FD-3234	—	—	—	—
Dichelobacter nodosus A1001	NSW, Australia	Ovine	A1001	+	—	+	—
Dichelobacter nodosus JIR3528	NSW, Australia	Ovine	JIR3528	—	+	—	+

a Tests were evaluated using genomic DNA extracted from 15 bacterial species. Virulent (*aprV2* positive) and benign (*aprB2* positive) D. nodosus control strains were also included.

b ID, identification.

The repeatability of the qPCR test developed by Frosth et al. (21) was evaluated for the *aprV2* and *aprB2* alleles with three concentrations of genomic DNA per reaction. The coefficients of variation (CVs) were similar (<5%) for each of the three DNA concentrations for both the *aprV2* and *aprB2* alleles.

The two qPCR tests were compared using 430 lesion swabs collected from 18 flocks (flocks 5 to 11, 17 to 18, and 24 to 32, as described in Table 1). The qPCR test developed by Frosth et al. (21) detected the *aprV2* allele in 48 lesion swabs and the *aprB2* allele in 26 lesion swabs that the test developed by Stäuble et al. (22) did not. Consequently, a decision was made to proceed with the assay developed by Frosth et al. (21) for a larger test evaluation.

### Comparison of clinical diagnosis and virulence tests at the flock level.

### *aprV2*/*B2* qPCR test.

The qPCR test was evaluated using 758 lesion swabs collected from 40 Australian sheep flocks. An outbreak was more likely to be classified as virulent with the qPCR test than as classified clinically (*P* < 0.0009) (Table 3). The level of agreement between field diagnosis and the qPCR test was fair (kappa statistic, 0.353). At the flock level, the diagnostic sensitivity (DSe) and diagnostic specificity (DSp) of the qPCR test were 100% and 31.3%, respectively. The level of agreement was considerably lower at the foot swab level (kappa statistic, 0.096) (Table 4), where DSe and DSp were 98.1% and 18.8%, respectively.

**TABLE 3:** Flock-level comparison of clinical diagnosis and *aprV2*/*B2* qPCR using 758 lesion swabs collected from 40 Australian sheep flocks^*a*^

Clinical diagnosis^*b*^	No. of flocks with a laboratory diagnosis (*aprV2/B2* qPCR) of:^*c*^		
	Benign	Virulent	Total
Benign	5	11	16
Virulent	0	24	24
Total	5	35	40

a McNemar's χ^2^ = 11.0, *P* = 0.0009; kappa statistic = 0.353 (95% CI, 0.105–0.601), DSe = 100% (95% CI, 87.5–100%), DSp = 31.3% (95% CI, 11.0–58.6%).

b Clinical diagnoses are given in Table 1.

c qPCR according to reference 21. Benign, swabs tested negative for the *aprV2* allele; virulent, ≥1 swab tested positive for the *aprV2* allele.

**TABLE 4:** Sample-level comparison of clinical diagnosis and the *aprV2*/*B2* qPCR using genomic DNA extracted directly from 758 foot swabs collected from 40 Australian sheep flocks^*a*^

Clinical diagnosis^*b*^	No. of foot swabs with a laboratory diagnosis (*aprV2/B2* qPCR) of:^*c*^		
	Benign	Virulent	Total
Benign	84	363	447
Virulent	6	305	311
Total	90	668	758

a McNemar's χ^2^ = 345.39, *P* < 0.0001, kappa statistic = 0.096 (95% CI, 0.064–0.128), DSe = 98.1% (95% CI, 95.9–99.3%), DSp = 18.8% (95% CI, 15.3–22.7%).

b Clinical diagnosis was at the flock level (Table 1).

c qPCR according to reference 21. Benign, swabs that tested negative for the *aprV2* allele; virulent, swabs that tested positive for the *aprV2* allele.

### Elastase test.

The elastase test was used to evaluate 469 D. nodosus isolates collected from 38 Australian sheep flocks (Table 5). There was no significant difference (*P* = 0.0833) between the proportion of outbreaks classified as virulent by clinical diagnosis or by the elastase test. The level of agreement between clinical diagnosis and the elastase test was almost perfect (kappa statistic, 0.822). At the flock level, the DSe and DSp of the elastase test were 100% and 78.6%, respectively.

**TABLE 5:** Flock-level comparison of clinical diagnosis and the elastase test using 469 D. nodosus isolates collected from 38 Australian sheep flocks^*a*^

Clinical diagnosis	No. of flocks with a laboratory diagnosis (elastase test) of:^*b*^		
	Benign	Virulent	Total
Benign	11	3	14
Virulent	0	24	24
Total	11	27	38

a Lesion swabs were not collected for microbiological culture from flocks 39 and 40. McNemar's χ^2^ = 3.0, *P* = 0.0833; kappa statistic = 0.822 (95% CI, 0.633–1.000), DSe = 100% (95% CI, 85.8–100%), DSp = 78.6% (95% CI, 49.2–95.3%).

b Benign, no D. nodosus isolates were elastase positive at ≤12 days; virulent, ≥1 D. nodosus isolate was elastase positive at ≤12 days.

Clinical diagnosis and the elastase test were also compared at the isolate level (Table 6). There was a significant difference (*P* < 0.0001) between the proportion of isolates from clinically virulent outbreaks that were elastase negative and those from clinically benign outbreaks that were elastase positive. The level of agreement was moderate (kappa statistic, 0.431). At the isolate level, DSe and DSp of the elastase test were 69.9% and 80.5%, respectively.

**TABLE 6:** Isolate-level comparison of clinical diagnosis and the elastase test using 469 D. nodosus isolates collected from 38 Australian sheep flocks^*a*^

Clinical diagnosis	No. of isolates with a laboratory diagnosis (elastase test) of:^*b*^		
	Benign	Virulent	Total
Benign	107	26	133
Virulent	101	235	336
Total	208	261	469

a Lesion swabs were not collected for microbiological culture from flocks 39 and 40. McNemar's χ^2^ = 44.29, *P* < 0.0001, kappa statistic = 0.431 (95% CI, 0.352–0.51), DSe = 69.9% (95% CI, 64.7–74.8%), DSp = 80.5% (95% CI, 72.7–86.8%).

b Benign, isolates that were elastase negative at ≤12 days; virulent, isolates that were elastase positive at ≤12 days.

### Gelatin gel test.

The gelatin gel test was used to evaluate samples collected from six clinically benign outbreaks (flocks 27, 28, 29, 30, 31, and 32). Overall, 49.1% (28/57) of the D. nodosus isolates obtained from these flocks were heat stable (virulent).

### Comparison of virulence tests at the isolate level.

### *aprV2*/*B2* qPCR test and elastase test.

There was a significant difference (*P* < 0.0001) between the results from the elastase test and those from the qPCR test. Only 52.7% (213/404) of D. nodosus isolates were classified as virulent by both tests (Table 7). There was considerable discrepancy between the elastase test and the qPCR test for isolates classified as benign by the elastase test, as 73.2% (139/190) of these isolates were classified as virulent by the qPCR test. These isolates were classified as benign as they were elastase negative at the cutoff point of 12 days; however, elastase activity was observed for 80.6% (112/139) of these isolates after 16 to 28 days of incubation. The level of agreement between the two tests was only fair (kappa statistic, 0.275).

**TABLE 7:** Comparison of the elastase test and the *aprV2*/*B2* qPCR test using 404 D. nodosus isolates obtained from 38 Australian sheep flocks^*a*^

Laboratory diagnosis (elastase test)	No. of isolates with a laboratory diagnosis (*aprV2*/*B2* qPCR) of:^*b*^		
	Benign	Virulent	Total
Benign	51	139	190
Virulent	1	213	214
Total	52	352	404

a Lesion swabs were not collected for microbiological culture from flocks 39 and 40. McNemar's χ^2^ = 137.00, *P* < 0.0001, kappa statistic = 0.275 (95% CI, 0.207–0.343).

b qPCR according to reference 21. Benign, isolates that were negative for the *aprV2* allele; virulent, isolates that were positive for the *aprV2* allele.

### *aprV2*/*B2* qPCR test and the gelatin gel test.

There was a significant difference (*P* < 0.0001) between the results from the gelatin gel test and those from the qPCR test, with 86.2% (25/29) of isolates classified as benign (unstable) by the gelatin gel test classified as virulent by the qPCR test (Table 8). The level of agreement between the two tests was poor (kappa statistic, 0.101, 95% confidence interval [CI] −0.024 to 0.244).

**TABLE 8:** Comparison of the *aprV2*/*B2* qPCR test and the gelatin gel test using 57 D. nodosus isolates obtained from flocks 27, 28, 29, 30, 31, and 32^*a*^

Laboratory diagnosis (gelatin gel test)	No. of isolates with a laboratory diagnosis (*aprV2*/*B2* qPCR) of:^*b*^		
	Benign	Virulent	Total
Benign	4	25	29
Virulent	1	27	28
Total	5	52	57

a All of these flocks presented with clinically benign footrot (see Table 1). McNemar's χ^2^ = 23.04, *P* < 0.0001, kappa statistic = 0.101 (95% CI, −0.024 to 0.244).

b qPCR according to reference 21. Benign, isolates that were negative for the *aprV2* allele; virulent, isolates that were positive for the *aprV2* allele.

### Elastase test and the gelatin gel test.

Paired elastase and gelatin gel test results were available for 56 D. nodosus isolates (Table 9). There was a significant difference between the results from the two tests (*P* < 0.0001), with 42.9% (21/49) of isolates classified as benign by the elastase test classified as virulent by the gelatin gel test. The level of agreement between the tests was slight (kappa statistic, 0.193).

**TABLE 9:** Comparison of the elastase test and the gelatin gel test using 56 D. nodosus isolates obtained from flocks 27, 28, 29, 30, 31, and 32^*a*^

Laboratory diagnosis	No. of isolates with a laboratory diagnosis (elastase test) of:^*b*^		
	Benign	Virulent	Total
Benign	28	1	29
Virulent	21	6	27
Total	49	7	56

a All of these flocks presented with clinically benign footrot (see Table 1). McNemar's χ^2^ = 18.18, *P* < 0.0001, kappa statistic = 0.193 (95% CI, 0.015–0.370).

b Benign, isolates that were elastase negative at ≤12 days; virulent, isolates that were elastase positive at ≤12 days.

## DISCUSSION

In this study, we undertook an evaluation of clinical diagnoses and microbial virulence tests with an emphasis on the qPCR tests developed by Stäuble et al. (22) and Frosth et al. (21), and we subjected the test developed by Frosth et al. (21) to a larger evaluation, in accordance with chapter 1.1.6 of the *Manual of Diagnostic Tests and Vaccines for Terrestrial Animals* (27), which states that a diagnostic test must be evaluated using appropriate reference samples from a defined target population to be declared fit for its intended purpose.

The analytical performance of the two qPCR tests was evaluated using a set of 430 samples collected from 18 Australian flocks. The test developed by Frosth et al. (21) detected the *aprV2* allele in some samples and the *aprB2* allele in other samples that the test developed by Stäuble et al. (22) did not. This discrepancy may have resulted from PCR inhibitors present in the DNA extract; Frosth et al. (21) include bovine serum albumin (BSA) in their reaction mixture, which has been shown to alleviate PCR inhibition (28). The lower sensitivity of the test developed by Stäuble et al. (22) may also be a consequence of variable primer binding, as there is known to be a single-nucleotide polymorphism (SNP) in the region of their forward primer in some D. nodosus strains (29). Consequently, we proceeded with a larger evaluation using the test developed by Frosth et al. (21).

Comparing the results from this qPCR test with clinical diagnoses, there was moderate to poor agreement at the flock and individual swab sample levels, and the diagnostic specificity of the qPCR was low overall (up to ∼30%).By contrast, there was much better agreement between clinical diagnoses and the results from the elastase test at the flock and individual sample levels with diagnostic specificity ∼80%. Isolate-level comparison of the qPCR test and elastase test revealed that 73.2% (139/190) of isolates that were deemed benign by the elastase test were deemed virulent by the qPCR test (*aprV2* positive). The elastase test depends on visual assessment of the digestion of elastin particles in an agar matrix, and a cut point based on incubation time is applied, generally 10 to 12 days beyond which an isolate is deemed benign (8, 12). Thus, the amount of elastase activity and the rate at which it is elaborated influence the outcome of the test. Elastase activity was observed for 80.6% of the benign isolates after further incubation, confirming temporal and quantitative variations in the expression of the AprV2 enzyme between strains. Regardless, the results indicate that some strains that possess *aprV2* did not express detectable elastase activity and may not be capable of inducing severe disease. Consequently, identification of the *aprV2* allele may not be a reliable indicator of virulence.

Clinical diagnoses in this study were made using objective criteria that have been applied successfully in a state-wide eradication program in NSW (30). However, these criteria ignore the true spectrum of severity that is possible in ovine footrot, which ranges from inapparent through mild to severe (7). Forcing a dichotomous clinical classification was pragmatic from the perspective of disease control and, in the present study, enabled the comparison with dichotomous laboratory test outcomes. The authors acknowledge that this may lead to some inaccurate classifications of both clinical and laboratory results given that the latter could also be continuous variables. Nevertheless, the trends are very obvious, and the discrepancies between clinical and laboratory diagnoses are substantial. In Fig. 2, the frequency of sheep with foot lesions in each score category are shown for 28 flocks. It is clear that laboratory diagnoses of virulent footrot do not match the clinical patterns in flocks in which there were no sheep with severe lesions and where there was sufficient history to be very confident that virulent footrot was not present. Test outcomes like this undermine confidence and may lead to farmers disengaging from programs to control the disease.

These findings elaborate those of Stäuble et al. (22) and Frosth et al. (21) who reported a high level of agreement between the qPCR test and clinical diagnosis. The case definitions used by these authors, which differ from those used in Australia, may partially explain this discrepancy. Stäuble et al. (22) did not classify outbreaks as clinically virulent or benign; rather, flocks were classified as “nonaffected” (all feet assigned a score of 0) or “affected” (one or more feet assigned a score ≥1). The authors report that all lesion swabs from “affected” flocks were positive for the *aprV2* allele and negative for the *aprB2* allele and that >80% of samples from the “nonaffected” flocks were positive for the *aprB2* allele. Therefore, the authors concluded that there was a high level of agreement between clinical diagnosis and the qPCR test. However, if the flocks are reclassified using the case definitions applied in Australia, at least two of the “affected” flocks would be regarded as having clinically benign footrot, as no score 4 lesions were observed in these flocks.

Similar discrepancies are apparent in the data provided by Frosth et al. (21). Each flock was assigned to one of four categories: (i) predominantly score 0 with some score 1 lesions, (ii) many score 1 lesions but no scores >1, (iii) at least one animal with a score 2 lesion, and (iv) at least one animal with a score 3 lesion. It is evident that under the Australian classification system, categories one and two could describe a flock with ovine interdigital dermatitis (OID), benign footrot, or the early stages of virulent footrot. Similarly, categories three and four could describe an outbreak of benign footrot or the early stages of an outbreak of virulent footrot. No category describes virulent footrot exclusively.

The clinical observations reported in this study support the use of a classification system that is based on the prevalence of score 4 lesions rather than the presence or absence of foot lesions of any grade. We observed that D. nodosus strains classified as benign by the qPCR test (*aprB2* positive) are capable of inducing severe, underrun lesions in a small proportion of susceptible sheep (Table 1), a finding in keeping with prior knowledge of phenotypic protease tests (13). However, at the flock level, the impact of foot disease in these flocks was minor. In flock 33, for example, 42/1,716 sheep presented with score 4 lesions, but the overall prevalence of sheep with foot lesions of any grade in this flock was low (Fig. 2), despite all sheep being exposed to the same strain under the same environmental conditions. Thus, it would be inappropriate to subject these flocks to the same regulatory activity (quarantine and compulsory disease control) as those deemed to have virulent footrot, as the D. nodosus strains present in these outbreaks would most likely be incapable of inducing severe disease in a large proportion of sheep. In general, benign footrot is not considered amenable to control, and attempts to do so would expose farmers to ongoing costly measures (26, 31). Culling susceptible sheep that develop severe lesions when infected with benign strains is the most practical course of action (32). The experience in Australia using conventional phenotypic virulence tests is that after a control program directed at virulent footrot, benign strains of D. nodosus persist in flocks (33–35).

The identification of *aprV2*-positive strains of D. nodosus in clinically healthy flocks provides further evidence that the *aprV2* may be an unreliable virulence marker. Stäuble et al. (22) reported that seven samples collected from “nonaffected” (clinically healthy) flocks were positive for the *aprV2* allele, alone or in combination with the *aprB2* allele. In Switzerland, Locher et al. (36) evaluated the qPCR test developed by Stäuble et al. (22) as a potential screening tool for identifying virulent D. nodosus isolates in clinically healthy flocks and reported that *aprV2*-positive isolates were identified in four flocks on one or more occasions, despite the flocks remaining clinically healthy for the duration of the study. This finding may also reflect differences in breed susceptibility, as the European breeds are inherently more resistant to footrot than the Merino breed (37).

In conclusion, strains of D. nodosus that may vary in virulence interact with other microbial flora on the skin of the foot after environmental predisposition and, depending on the inherent susceptibility of the host, may then induce dermatitis and degradation of the hoof. This complex interplay between the pathogen, the environment, and the host creates a difficult set of circumstances for diagnosticians. Nevertheless, virulence of D. nodosus at least partially depends on the elaboration of a protease coded by *aprV2* and manifests as elastase activity. In this study, we demonstrated that *aprV2*-positive D. nodosus isolates are frequently isolated from outbreaks of clinically benign footrot and that phenotypic evidence of elastase activity was more closely related to clinical diagnosis than was the mere presence of the gene. As benign footrot is not associated with significant animal welfare concerns, and as attempts to control benign footrot are both expensive and ineffective, we conclude that the qPCR test is not fit for its intended purpose. There is a considerable risk that producers would be subject to unnecessary and costly regulatory activity if the *aprV2*/*B2* qPCR was used as the sole basis for diagnosis. In this study, we demonstrated that there is a diversity of phenotypes among D. nodosus isolates that possess the *aprV2* allele, though the basis of this diversity is currently unknown. We recommend further investigation of the molecular basis of virulence.

## MATERIALS AND METHODS

### Flock selection.

Forty Australian sheep flocks were included in this study from target populations in southern Australia, including 24 flocks with clinically virulent footrot and 16 flocks with clinically benign footrot (Table 1). Lesion swabs were collected from flocks 1 to 32 and 34 to 40 between June 2014 and August 2016 for diagnostic purposes. Lesion swabs were collected from flock 33 in 2006 during the course of a previous study (38).

### Clinical examination and diagnosis.

Three methods of clinical diagnosis were used during this study as shown in Table 1. The method depended on whether the sheep were examined by the authors or an animal health officer (AHO), on the number of sheep or mobs examined, and on prior diagnostic investigations. On each farm, sheep were placed in dorsocaudal recumbency, and each foot was carefully examined, as described by Stewart and Claxton (8). A score was assigned to each foot according to the scoring system devised by Egerton and Roberts (10). This is a standardized systematic approach to grading footrot lesions, with a high level of repeatability (39). All observers were trained and highly experienced with foot scoring. For methods 1 and 2, the diagnosis of clinically virulent and benign footrot was based on the prevalence of score 4 lesions observed in ≥100 sheep selected from the flock by systematic random sampling, as described by Egerton (11), or after the producer had drafted off a proportion of the flock or mob as a convenience sample. A mob is a subset of a flock run separately for management purposes. To align with the dichotomous classification system used by regulatory authorities, outbreaks were classified as clinically benign when score 4 lesions were observed in <10% of the flock or mob or clinically virulent when score 4 lesions were observed in ≥10% of the flock or mob. This dichotomous system is pragmatic and was used during the NSW Footrot Strategic Plan to identify farms for the application of compulsory control and eradication measures, resulting in a reduction in the prevalence of farms affected with virulent footrot from 15% to <1% (30).

### Method 1.

At least 100 sheep were examined by the authors or an AHO. The producer presented either (i) one mob for examination because it was the only one with clinical footrot on the farm or it was the mob with the most severe clinical signs of lameness or (ii) two mobs for examination (50 inspected per mob) because clinical signs of lameness were previously observed in both mobs or foot lesions were previously observed in both mobs during routine husbandry procedures. A flock history was provided by the producer at the time of inspection. Lesion swabs were collected at the time of examination by the authors.

In all flocks that appeared to have clinically benign footrot, additional criteria were used to support the diagnosis. (i) The flock/mob must have been examined previously on two or more occasions by the authors or an experienced AHO, according to the system described by Stewart and Claxton (1993). The disease must have been classified as clinically benign on each occasion, according to the system of Egerton (11). The retrospective foot score data were inspected by the authors. (ii) Environmental conditions must have been favorable for the transmission and expression of the disease in the 2 weeks prior to each of the examinations (average daily air temperature ≥10°C [50°F], consistent rainfall) (6). Climatic data were obtained from the nearest Bureau of Meteorology (BOM) weather station. (iii) The flock history was obtained from the producer and did not suggest clinically virulent footrot. There was no clinical evidence of virulent footrot having been present in the flock previously, i.e., old lesions (such as damage to the abaxial hoof wall indicative of underrun lesions having been present) were not observed. (iv) Topical treatments that may suppress or mask the severity of disease, such as antiseptic foot bathing, had not been used in the 4 weeks preceding each examination. (v) The sheep were all Merino, which are naturally susceptible to footrot (37).

### Method 2.

A small number of animals were examined by an experienced AHO for the purpose of collecting lesion swabs. The sheep were sampled when convenient after the producer had drafted-off a proportion of the flock. The flock had been examined by the same AHO on two or more previous occasions and a clinical diagnosis made using method 1. As such, there was an interval between the time at which the clinical diagnoses were made and the time at which the lesion swabs were collected. The AHO informed the authors of his or her clinical diagnoses but did not provide the retrospective foot score data.

### Method 3.

Sheep in a “hospital mob” were examined by the authors or an experienced AHO. Between 10 and 60 sheep from each hospital mob were examined on each farm, as indicated in Table 1. The sheep were sampled when convenient after the producer had drafted-off a proportion of the flock or the entire mob was sampled. The sheep were separated from the parent flock(s) by the producer because they had the most severe clinical signs of lameness or because they were the only sheep in the parent flock(s) with foot lesions. The sheep had not been examined previously by the authors or an experienced AHO, and retrospective foot scores were not available. However, a flock history was obtained from the producer describing the progression of the disease since it was first introduced to the flock. Clinical diagnosis was based on the severity of clinical disease observed in the hospital mob, on the number of sheep with score 4 lesions separated from the parent flocks(s), on the size of the parent flock (and therefore a rough estimate of apparent prevalence of sheep with severe lesions was possible), and on the flock history. Lesion swabs were collected at the time of examination by the authors or an AHO.

### Collection of lesion swabs.

The interdigital skin or the active margin of an underrun lesion was swabbed with a sterile, cotton-tipped swab (CLASSIQSwabs, Copan Italia, Italy). In most cases, two swabs were collected from each lesion: the first was placed into a 5-ml serum vial containing modified Stuart's transport medium (mSTM) for microbiological culture, and the second was placed into a 1.5-ml microcentrifuge tube containing 500 μl of a lysis solution (buffer RLT [Qiagen] or nuclei lysis solution [Promega]) for DNA extraction and direct (culture-independent) testing. All swabs were transported on ice. Swabs collected for microbiological culture were processed immediately upon receipt at the laboratory. Swabs collected for direct testing were stored at 4°C prior to DNA extraction, which was undertaken 24 to 48 h after receipt.

### Microbiological culture of D. nodosus.

Upon receipt at the laboratory, each lesion swab was removed from the mSTM and cultured anaerobically, as described previously (15).

### Archival samples.

Lesion swabs were collected from flock 33 in 2006. The entire flock had been inspected on several occasions over a period of 3 years as part of a previous study (38), and the disease was classified as clinically benign on each occasion based on method 1. Individual D. nodosus isolates obtained in 2006 had been freeze-dried and stored at 4°C. For the present study, 15 randomly selected freeze-dried isolates were reconstituted in 100 μl phosphate-buffered saline (PBS) and spread-plated onto 4% hoof agar (HA) for microbiological culture, as described previously (15).

### Control strains.

Virulent D. nodosus type strain A1001 and benign D. nodosus field strain JIR3528 were used as virulent (*aprV2* positive) and benign (*aprB2* positive) control strains, respectively.

### DNA extraction.

Each pure culture of D. nodosus was harvested with a cotton-tipped swab, and DNA was extracted using the Wizard genomic DNA purification kit (Promega, WI, USA) in accordance with the protocol for Gram-negative bacteria. Extraction of DNA from lesion swabs was undertaken using a magnetic bead DNA purification kit (BioSprint 96 one-for-all vet kit, Qiagen) according to the BS96 Vet 100 protocol.

### PCR identification of D. nodosus.

D. nodosus was identified via conventional PCR or real-time PCR amplification of a variable region of the D. nodosus 16S rRNA gene (40, 41). PCR products were visualized on 2% agarose gels stained with RedSafe (iNtRON Biotechnology, South Korea) and viewed under UV light, as described previously (15).

### Phenotypic virulence testing.

### Gelatin gel test.

Each pure culture of D. nodosus was evaluated using the gelatin gel test, as described previously (12). Known stable (A1001) and unstable (JIR3528) D. nodosus strains were included as controls.

### Elastase test.

Each pure culture of D. nodosus was evaluated for elastase activity, as described previously (13). An isolate with known elastase activity (virulent D. nodosus type strain A1001, elastase positive at 4 to 8 days postinoculation) was included as a virulent control for each test.

### *aprV2*/*B2* qPCR test.

Primers, probes, and master mixes reported by Stäuble et al. (22) and Frosth et al. (21) were ordered from Thermo Fisher Scientific, Inc. Amplification was performed in a Stratagene Mx3000P thermocycler (Agilent Technologies, Santa Clara, CA). Reaction mixtures and cycling conditions were as described by Stäuble et al. (22). Reaction mixtures as described by Frosth et al. (21) were used; however, as the thermocycler used in this study was unable to accommodate the fast-cycling conditions described by the authors, the cycling conditions recommended in the TaqMan gene expression master mix protocol were used, consisting of a UNG activation step of 2 min at 50°C, an initial denaturation step of 10 min at 95°C, followed by 40 cycles of denaturation at 95°C for 15 s, and annealing/extension at 60°C for 60 s.

Virulent (*aprV2* positive) and benign (*aprB2* positive) controls were included in each run. A valid qPCR run was one in which: (i) there was amplification of both replicates of the *aprV2* and *aprB2* positive controls, with threshold cycle (*C_T_*) values falling within the range of the standard curve (0.005 pg to 5,000 pg); (ii) there was no amplification of the *aprV2* and *aprB2* negative controls; and (iii) given that the qPCR test was only used to determine the presence or absence of the two alleles during this study, amplification efficiencies of 85 to 110% were accepted. The fluorescence threshold was initially set automatically for each run by the MxPro software (Agilent Technologies, Santa Clara, CA). However, to ensure that comparable *C_T_* values were calculated for each run, the average fluorescence threshold was calculated for each target using the fluorescence threshold values set for all 20 runs, and applied retrospectively to each run.

### Analytical performance of the qPCR tests.

### Analytical sensitivity.

Amplification of the *aprV2* and *aprB2* alleles was analyzed separately using serial dilutions of genomic DNA prepared from pure cultures of virulent D. nodosus type strain A1001 and benign D. nodosus field strain JIR3528, respectively. The limits of detection (LOD) and amplification efficiencies were calculated for the *aprV2* and *aprB2* alleles for both qPCR tests. Data were collected from 20 individual experiments, with each reaction performed in duplicate (*n* = 40 data points per concentration). DNA template concentrations ranged from 0.0005 pg to 5,000 pg of D. nodosus genomic DNA per reaction. The LOD was defined as the lowest concentration of genomic DNA at which amplification occurred for 50% of the replicates (27).

### Analytical specificity.

The analytical specificity of each qPCR test was evaluated using DNA extracted from 15 bacterial species along with the virulent and benign D. nodosus type strains (Table 2).

### Repeatability.

The between-run repeatability of the qPCR test was determined for the *aprV2* and *aprB2* alleles using three different concentrations of D. nodosus genomic DNA: 5,000 pg, 50 pg, and 0.5 pg per reaction. The coefficient of variation (CV) was calculated for each concentration using *C_T_* values collected across 20 qPCR runs, with each reaction performed in duplicate (*n* = 40 data points per concentration). The coefficient of variation (CV) was calculated for each concentration of genomic DNA as CV = standard deviation of replications/mean of replicates × 100.

### Diagnostic performance of each virulence test.

The levels of agreement between clinical diagnoses and laboratory diagnoses of virulent and benign footrot using each laboratory virulence test were compared. An outbreak of footrot was classified as virulent if one or more isolates were classified as virulent by a given laboratory virulence test.

### Diagnostic sensitivity and specificity.

### (i) qPCR test.

At the flock level, diagnostic sensitivity (DSe) was defined as the percentage of clinically virulent flocks in which one or more swabs tested positive for the *aprV2* allele, while diagnostic specificity (DSp) was defined as the percentage of clinically benign flocks in which none of the swabs tested positive for the *aprV2* allele. At the foot swab level, DSe was defined as the percentage of foot swabs collected from sheep in clinically virulent flocks that tested positive for the *aprV2* allele, while DSp was defined as the percentage of foot swabs collected from clinically benign flocks that tested negative for the *aprV2* allele.

### (ii) Elastase test.

At the flock level, DSe was defined as the percentage of clinically virulent flocks from which one or more elastase-positive D. nodosus isolates were obtained, while DSp was defined as the percentage of clinically benign flocks from which no elastase-positive isolates were obtained. At the isolate level, DSe was defined as the percentage of isolates obtained from clinically virulent flocks that were elastase positive, while DSp was defined as the percentage of isolates obtained from clinically benign flocks that were elastase negative.

### (iii) Gelatin gel test.

DSp was evaluated at both the flock and isolate levels. At the flock level, DSp was defined as the percentage of clinically benign flocks from which only heat-labile D. nodosus isolates were obtained. At the isolate level, DSp was defined as the percentage of isolates obtained from clinically benign flocks that were heat labile.

### Statistical analysis.

The levels of agreement between clinical and laboratory diagnoses were evaluated using Cohen's kappa statistic (42). Kappa statistics were interpreted using the standards for strength of agreement proposed by Landis and Koch (43): ≤0, poor agreement; 0.01 to 0.20, slight agreement; 0.21 to 0.40, fair agreement; 0.41 to 0.60, moderate agreement; 0.61 to 0.80, substantial agreement; and 0.81 to 1.00, almost perfect agreement. McNemar's chi-square test for paired observations (44) was performed to establish if there were statistically significant differences between the proportions of outbreaks classified as virulent by clinical or laboratory diagnosis using each of the three virulence tests. The results of each individual laboratory virulence test were also compared using this approach. All statistical analyses were conducted in Microsoft Excel 2010. Exact 95% binomial confidence intervals were calculated for diagnostic sensitivities and specificities in GenStat 16th Edition (VSN International, UK).
